# Group B streptococcal screening, intrapartum antibiotic prophylaxis, and neonatal early-onset infection rates in an Australian local health district: 2006-2016

**DOI:** 10.1371/journal.pone.0214295

**Published:** 2019-04-04

**Authors:** Kathryn Braye, Maralyn Foureur, Koert de Waal, Mark Jones, Elise Putt, John Ferguson

**Affiliations:** 1 Faculty of Health, University of Technology, Sydney, New South Wales, Australia; 2 Hunter New England Health, Newcastle, New South Wales, Australia; 3 School of Nursing and Midwifery, University of Newcastle, Newcastle, New South Wales, Australia; 4 Department of Neonatology, John Hunter Hospital, Newcastle, New South Wales, Australia; 5 School of Biomedical Sciences and Pharmacy, University of Newcastle, Newcastle, New South Wales, Australia; 6 Hunter Medical Research Institute, Newcastle, New South Wales, Australia; 7 New South Wales Health Pathology, Newcastle, New South Wales, Australia; Monash University, AUSTRALIA

## Abstract

**Background:**

Intrapartum antibiotic prophylaxis (IAP) to reduce the likelihood of neonatal early-onset group B streptococcal infection (EOGBS) has coincided with major reductions in incidence. While the decline has been largely ascribed to IAP following either universal screening or a risk-based approach to identify mothers whose babies may most benefit from IAP, there is lack of high quality evidence to support this view.

**Aims:**

To describe management of maternal GBS colonisation in one local health district using universal screening and assess rates of EOGBS over time.

**Methods:**

A retrospective cohort study was undertaken to describe compliance with GBS management, to determine the incidence of EOGBS and association between rates and maternal screening. Linking routinely collected maternity and pathology data, we explored temporal trends using logistic regression and covariates for potential effect modifiers.

**Results:**

Our cohort included 62,281 women who had 92,055 pregnancies resulting in 93,584 live born babies. Screening occurred in 76% of pregnancies; 69% had a result recorded, 21.5% of those were positive for GBS. Prophylaxis was used by 79% of this group. Eighteen babies developed EOGBS, estimated incidence/1000 live births in 2006 and 2016 was 0.35 (95% CI, 0.07 to 0.63) and 0.1 (95% CI, 0 to 0.2) respectively. Seven of 10 term babies with EOGBS were born to mothers who screened negative. Data were unable to provide evidence of difference in rates of EOGBS between screened and unscreened pregnancies. We estimated the difference in EOGBS incidence from crude and weighted models to be 0 (95% CI, -0. 2 to 0.17) and -0.01 (95% CI, -0.13 to 0.10) /1000 live births respectively.

**Conclusion:**

No change was detected in rates of EOGBS over time and no difference in EOGBS in babies of screened and unscreened populations. Screening and prophylaxis rates were modest. Limitations of universal screening suggest alternatives be considered.

## Introduction

Early-onset group B streptococcal infection (EOGBS) is a high impact event that, despite its low frequency, remains a significant cause of early infant morbidity and mortality [1]. To reduce the likelihood of EOGBS, intrapartum antibiotic prophylaxis (IAP) was introduced in the 1980s and offered to women whose babies were thought to be most at risk. In the United States of America (USA), widespread use of IAP coincided with a decline in reported EOGBS rates; from 0.7/1000 live births in 1997 [2] to 0.22/ 1000 in 2016 [3]. However, since the pre-prevention era, the proportion of women and babies exposed to IAP has more than doubled (from 12% to 30%) in the USA and other high-income countries [4].

Antibiotics have saved millions of lives, but they are not without risk. Most recently concerns have been raised about the possible link between IAP exposure and dysbiosis of the infant’s founding microbiome, which may lead to adverse health effects in later life [5–9]. Research which highlights benefits, risks and limitations of GBS screening and IAP provision is therefore warranted.

### Background

#### Neonatal group B streptococcal colonisation and infection

Prior to the implementation of screening and IAP provision, it was believed that up to 50% of babies born vaginally to mothers with GBS colonisation would be colonised by the bacterium as part of their founding microbiome. Most of these babies were not compromised by GBS colonisation and remained well [10, 11]. In the absence of IAP, it is reported that 1–3% of babies colonised with GBS will develop EOGBS [12]; however, this proportion is difficult to quantify in the era of widespread IAP. In a global systematic review and meta-analysis, the incidence of EOGBS was 0·43/ 1000 live births (95% CI, 0·37–0·49) and global case fatality 12·1%, (6·2–18·3) [1].

#### Screening approaches

In 1996 the centers for disease control and prevention (CDC) published guidelines recommending that clinicians select women whose babies may benefit from IAP and offer prophylaxis to reduce the likelihood of EOGBS. The selection criteria were based on certain risk factors including maternal recto-vaginal GBS colonisation, rupture of membranes (ROM) ≥18 hours, intrapartum fever and prematurity [12–14]. History of bacteriuria in the index pregnancy and having a sibling diagnosed with EOGBS are also risk factors [12, 13].

In 2002, based on a large retrospective study in the USA [15], the CDC recommended universal screening for vaginal and rectal GBS colonisation of all pregnant women at 35–37 weeks’ gestation as the best method for GBS management [16]. When GBS status was unknown, a risk-based based approach for IAP was recommended. In 2010 the CDC continued to recommend universal screening [12] although globally, countries remain divided regarding optimal GBS management.

In Australia the evolution of GBS management strategies began in the late 1970s, based on the observation of an unexpectedly large number of EOGBS reported in one city. As a consequence of a review into local EOGBS rates in a large metropolitan Melbourne hospital, policy recommended a universal GBS screen for pregnant women and provision of IAP to those at risk [17]. This review influenced GBS management throughout the country. However Australia has never had a national GBS policy and Australian states and territories recommend different approaches for selecting women for IAP. Queensland, for example, recommends a risk-based approach [18] and NSW recommends either universal screening or a risk-based approach [19]. The latest guidelines from the Royal Australian and New Zealand college of Obstetricians and Gynaecologists (RANZCOG) [13] also recommend either approach. Conversely, New Zealand has undertaken local research [20–22] and continues to offer a risk-based approach to manage GBS risk. A recent Australian systematic review concluded that the odds of EOGBS in infants of any gestation were significantly lower with universal screening compared with risk-based screening (OR 0.45, 95% CI, 0.37–0.53). However the authors noted the quality of the studies critiqued was low [23].

#### Incidence of early-onset group B streptococcal infection

Reported rates of neonatal EOGBS vary markedly, particularly in areas with limited access to laboratory diagnosis. Variation in rates may reflect changes in reporting of cases and/or natural fluctuation, a true increase or decrease in incidence, or less than optimal implementation of prevention strategies. Rates of EOGBS are often reported on a voluntary basis and therefore may not represent all confirmed cases. Our data include live births only. Although it is probable that GBS was a contributing factor in a proportion of stillborn babies in our district [24], it was not possible to obtain data on these babies.

Reported live birth rates of EOGBS in the USA, and other high-income countries including Australia, have remained stable for nearly two decades, at below 0.5/1000 live births [12, 25, 26]. Exceptions include New Zealand where researchers compared 1998–99 EOGBS rates which were estimated at 0.5/1000 live births (95% CI, 0.38, 0.65) [22] to rates five years later after instituting a national consensus risk-based approach. In 2009–11 EOGBS rates had halved to 0.26/1000 live births (95% CI, 0.18–0.37) [21]. Other countries have reported an increase in rates. The UK from 0.48/1,000 live births (95% CI, 0.43 to 0.53) in 2000–2001 to 0.57/1,000 live births in 2014/2015 (95% CI, 0.52–0.62) and [14] the Netherlands 0·11/1000 live births to 0·19/1000 live births (p<0·0001.)[16].

#### Local practice

In 2005, our local health district, now called Hunter New England Local Health District (HNELHD), changed GBS management from identification of risk factors to universal culture based screening and provision of IAP in line with the CDC guidelines of the time [16]. A local study, reported a dramatic decline in EOGBS (84%) when universal screening was employed to select candidates for IAP. The study reported that to prevent one case of the infection 5,704 women needed to be screened and 1,911 women with a positive GBS result would be required to have IAP [27].

The regime for IAP was set locally at 1.2-grams of penicillin followed by 600mg four hourly until birth [28] and, due to our very low EOGBS rates, this regime has not changed despite the Australian therapeutic guidelines [29] and CDC [30] recommendations of 3-grams of penicillin followed by 1.5–1.8 grams four hourly until birth. Over a decade has passed since this local study and the change from a risk-based approach to universal screening. We were interested to assess compliance with universal GBS screening and IAP protocols and EOGBS rates in this population.

### Aims

To describe compliance with GBS management in an era of universal screening and to assess rates of neonatal EOGBS over time in a diverse Australian local health district.

## Methods

### Study setting and population

A retrospective cohort study was employed using data from pregnancies that resulted in live born babies in the Hunter New England local health district, New South Wales (NSW) Australia, over the period 2006–2016.

The study population included women whose pregnancies resulted in live born babies birthing in all publicly funded maternity services within HNELHD and their babies. The term “pregnancies” or “women whose pregnancies” is used in this paper as around one third of women had more than one pregnancy during the study period. Included births occurred in hospitals, alongside and freestanding birth centres and at home, between 1^st^ January 2006 and 31^st^ December 2016 Table 1.

**Table 1:** Pregnancies resulting in live born babies per unit 2006–2016.

Birthing unit	Pregnancies	Babies
John Hunter Hospital	41946	42964
Maitland	17285	17472
Tamworth	8058	8194
Manning	6379	6459
Armidale	3849	3913
Inverell	2283	2314
Muswellbrook	2180	2184
Belmont Midwifery Group Practice	1996	1996
Singleton	1795	1795
Moree	1708	1714
Gunnedah	1628	1628
Narrabri	1234	1235
Scone	880	882
Glen Innes	662	662
Gloucester	121	121
Manilla	51	51
TOTALS	92,055	93,584

Information concerning babies and their mothers was obtained from the maternity ObstetriX database and the NSW Health Pathology database (Auslab). ObstetriX (now e. Maternity) is a state wide surveillance system providing point-of-care data collection across antenatal, intrapartum and immediate postnatal periods. Clinicians contribute information soon after birth. The database is maintained by local health district (LHD) data custodians. The medical records of babies affected by EOGBS and their mothers were also scrutinised. Provision of IAP was documented in the medical record with two clinicians signing for receipt and timing of the medication.

We collected data on maternal antenatal and intrapartum risk factors, together with neonatal outcomes for the 18 babies with confirmed EOGBS. Maternal GBS colonisation, prematurity, ROM≥18 hours and maternal age were collected and used in analysis. While intrapartum fever, history of maternal GBS bacteriuria and history of a previous child with EOGBS are risk factors, and therefore considered in a decision to offer IAP, we were unable to obtain information on these variables at a population level.

### Microbiological cultures

Neonatal EOGBS can be defined as culture proven GBS bacteria found in a normally sterile site; either blood, causing sepsis or cerebrospinal fluid (CSF) causing meningitis, or both [31]. Researchers use a range of time frames to define early-onset; from 48 hours to 7 days post birth. We applied the definition used by the National Institute for Health Care and Excellence (NICE) guidelines. This guideline defines EOS as sepsis occurring ≤72 hours after birth [31].

Neonatal cultures positive for GBS were accessed from the NSW health pathology database used for most public health pathology across HNELHD. Blood and CSF culture data were also accessed from 3 of 4 private providers who service small facilities in the north-western region of HNELHD.

### Gestation and eligibility for group B streptococcal screening

Term gestation was defined as ≥37 weeks gestation, preterm <37 weeks gestation. Eligibility for GBS screening applied to all women whose pregnancies were ≥35 weeks gestation, which includes a small number of women whose pregnancies were preterm. Pregnancies that reached ≥35 weeks but <37 weeks gestation were classified as “eligible preterm pregnancies”. Screening should occur within five weeks of birth to maximise accuracy [26].

### Definition of screened and not screened

Identification of women whose pregnancies were screened or not screened required the combination of several fields within the obstetric database. Eligible pregnancies were regarded as “screened” if they met either of two categories: “screened with a result” available intrapartum or at ROM (n = 60,674 69%) or “Screened with no result” where screening results were not available or pending at the time of birth or ROM. Women whose pregnancies were regarded as “not screened” occurred if there was no entry in the ObstetriX database or a text entry that stated either “screening declined” or “not screened”.

### Definition of adequate intrapartum antibiotic prophylaxis

Intrapartum antibiotic prophylaxis was defined as adequate when the initial dose of IAP was given at least four hours prior to birth in line with current CDC and RANZCOG guidelines [12, 13].

### Mortality and morbidity

Live status (as of December 2017) for each baby who had experienced an EOGBS event was derived from the HNELHD patient demographics system linked to NSW death registration data. Admission and short-term morbidity were reported as serious or not serious. Serious morbidity was defined as the need for significant respiratory support requiring neonatal intensive care; or circulatory support and/or encephalopathy or seizures. It was not possible to assess long-term morbidity in our study.

### Ethics approval

The study was deemed by the chair of Hunter New England (HNE) human research ethics committee (HREC) not to require formal approval by the ethics committee. The study conforms to the obligations of the provision of privacy and confidentiality of patient data and clinical information, including NSW Health records and Information Privacy Act 2002 as requested in our letter of approval from HNE research Ethics and Governance unit. University of Technology Sydney, HREC, ratified this decision. No. 2014000115. Data were de-identified for the purposes of this study. Individual consent was not required.

### Statistical analysis

Descriptive statistics for women, their pregnancies, and live born babies are provided Table 1. Early-onset GBS incidence rates were calculated as events/1000 live births per year. We explored EOGBS in the babies of all women whose pregnancies reached ≥35 weeks gestation and were therefore eligible for GBS screening. Given the low number of EOGBS events we report both crude and inverse probability weighting to balance groups. The inverse probability weights were estimated using a separate logistic regression model with screening status as the outcome regressed on variables plausibly related to EOGBS and/or screening including gestation, birth weight, positive maternal GBS screening, ROM ≥18 hours and maternal age at each pregnancy (categorical indicating <20 years or all others). We also used logistic regression to model trends in EOGBS incidence over time. All models were checked for calibration and discrimination and we used a conventional significance level of 0.05 throughout.

## Results

### Study population

Sixteen publicly funded birthing units were included Table 1 ranging from one metropolitan facility with an alongside birth centre and an associated freestanding birth centre nearby (in total around 4000 births per year), several regional units (700 to 1500 births per year) through to small rural units (<250 births per year).

**Fig 1:**
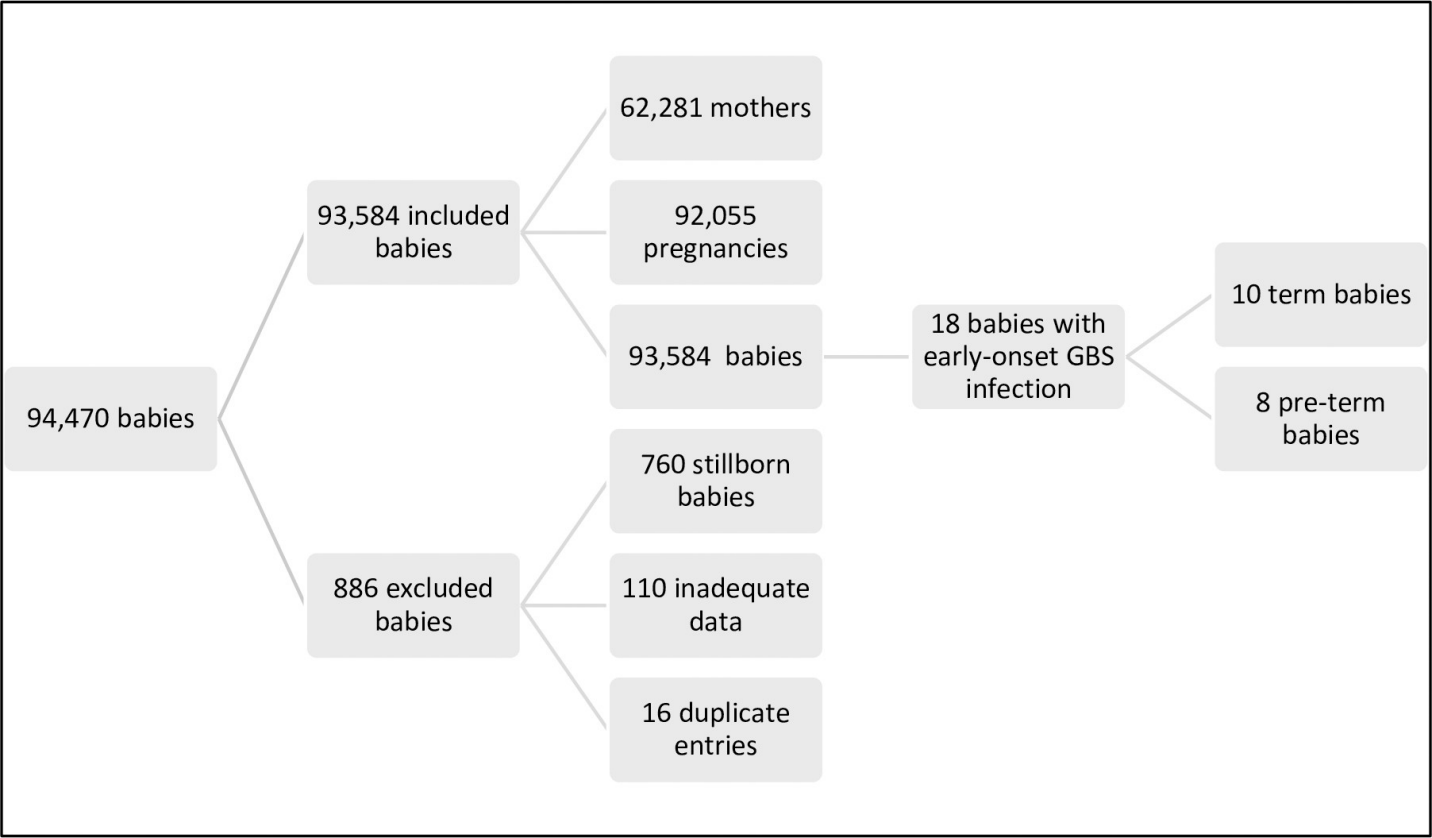
Inclusions and exclusions. EOGBS = early-onset group B streptococcal infection.

After exclusions, (babies who were stillborn and entries with inadequate or duplicate data) the study population included 62,281 women who had 92,055 pregnancies over the study period resulting in 93,584 live born babies. Ninety-eight per cent of babies (90,510) were singletons and 9.7% (9,146) of babies were preterm. Sixty-five babies had confirmed EOS. We found 18 babies with EOGBS, 10 term and eight preterm (0.19/1000 live births) Fig 1. Half of the term babies with EOGBS were born in the metropolitan unit and half in regional units. One was transferred from a regional unit to a higher level of care. All preterm babies with EOGBS were born at the metropolitan unit.

### Maternal GBS screening, colonisation and antibiotic prophylaxis

Nearly all women (96%) in our study had pregnancies ≥ 35 weeks and therefore were eligible for GBS screening. Seventy-six per cent of those eligible were reported to have a GBS screen. Of those, 69% had a result recorded in the database and 21.5% of those pregnancies were positive for GBS Table 2. Antibiotic prophylaxis was received by 79% of these women. Rates of positive maternal GBS colonisation in the cohort neither changed significantly from year to year nor materially between 2006 and 2016 Fig 2. Twelve per cent of women whose pregnancies were reported as GBS negative also received IAP Table 2. Reasons for administration of IAP to these women were not collected. Whether adequate IAP was given (≥4 hours before birth) could not be determined at a population level but was identified in individual cases.

**Fig 2:**
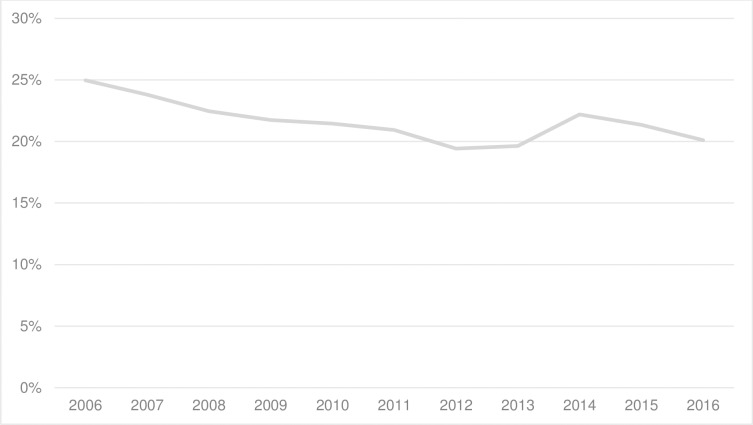
Maternal GBS colonisation.

**Table 2:** Eligible pregnancies, maternal GBS screening and colonisation rates, and IAP provision.

Birthing unit	Eligible pregnancies	Eligible pregnancies Screened (%)	Result recorded Pos/Neg (%)	GBS pos (%)	IAP given for GBS pos pregnancies (%)	IAP given for eligible not screened pregnancies (%)
**John Hunter Hospital**	38,885	32,011 (82)	29,529 (76)	7,089 (24)	5,600 (79)	825 (12)
**Maitland**	17,051	11,494 (67)	10,954 (64)	2,407 (22)	1,918 (80)	527 (9)
**Tamworth**	7,873	5,178 (66)	4,764 (61)	827 (17)	695 (84)	390 (14)
**Manning**	6,235	4,615 (74)	3,453 (55)	632 (18)	501 (79)	156 (10)
**Armidale**	3,781	2,411 (64)	2,148 (57)	401 (19)	359 (90)	202 (15)
**Inverell**	2,257	1,918 (85)	1,584 (70)	272 (17)	227 (83)	67 (20)
**Muswellbrook**	2,167	1,885 (87)	1,856 (86)	353 (19)	280 (79)	35 (12)
**Belmont Midwifery Group Practice**	1,996	1,479 (74)	1,417 (71)	297 (21)	24 (8)	4 (1)
**Singleton**	1,785	1,403 (79)	1,222 (68)	216 (18)	193 (89)	45 (12)
**Moree**	1,684	1,318 (78)	1,036 (62)	95 (9)	88 (93)	111 (30)
**Gunnedah**	1,621	1,432 (88)	996 (61)	164 (16)	148 (90)	66 (35)
**Narrabri**	1,223	816 (67)	583 (48)	64 (11)	44 (69)	71 (17)
**Scone**	876	673 (77)	646 (74)	140 (22)	127 (91)	27 (13)
**Glen Innes**	660	436 (66)	366 (55)	82 (22)	78 (95)	49 (22)
**Gloucester**	121	100 (83)	91 (75)	15 (16)	13 (87)	7 (33)
**Manilla**	51	33 (65)	29 (57)	4 (14)	3 (75)	0 (0)
**TOTALS**	88,266	67,202 (76)	60,674 (69)	13,058 (22)	10,298 (79)	2,582 (12)

Eligible pregnancy≥35 weeks gestation, Pos = positive, Neg = negative, IAP = intrapartum antibiotics prophylaxis

**Fig 3:**
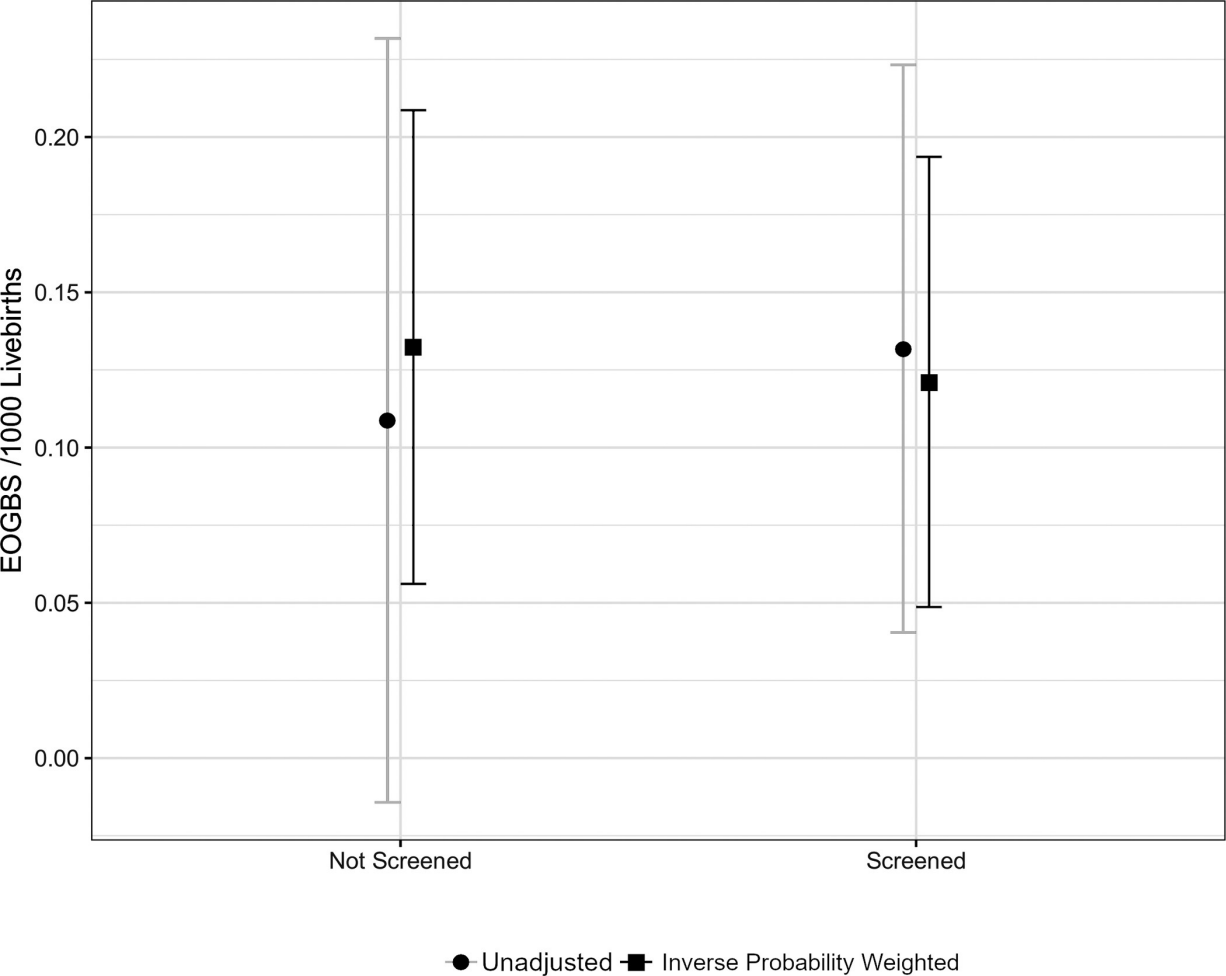
Screened versus unscreened pregnancies and rates of EOGBS. EOGBS = early-onset group B streptococcal infection.

None of our models gave evidence that the screened and unscreened cohorts had differing rates of EOGBS Fig 3. We estimated the difference in EOGBS incidence across reported screening status from the crude and weighted models to be 0 (-0.2 to 0.17) /1000 live births and -0.01 (95% CI, -0.13 to 0.10) /1000 live births respectively. Adjusting for a temporal trend did not materially impact the estimates.

### Early-onset group B streptococcal infection over time

**Fig 4:**
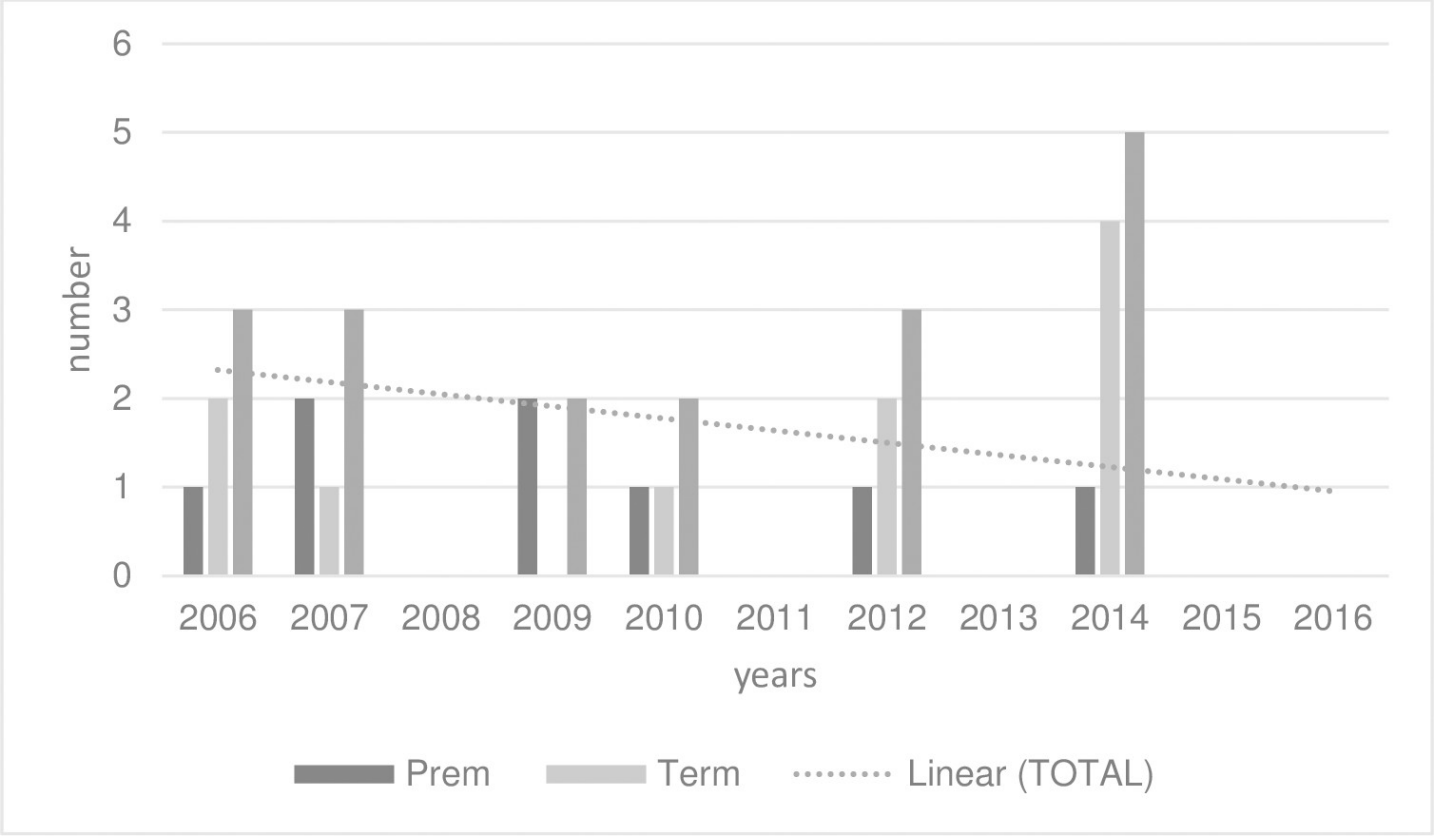
Incidence of term and preterm EOGBS 2006–2016.

The odds ratio for the annual temporal trend of EOGBS obtained from the exponentiated parameter estimates was 0.88 (95% CI, 0.75 to 1.03, p = 0.11). Model estimates for incidence per /1000 live births in 2006 and 2016 were 0.35 (95% CI, 0.07 to 0.63) and 0.1 (95% CI, 0 to 0.2) respectively Fig 4. A bootstrapped estimate for the difference between the 2006 and 2016 incidence of EOGBS was -0.28 (95% CI, -0.04 to 0.74) suggesting negligible support for a change even over the 10-year interval.

### Eligible babies with early-onset group B streptococcal infection

Ten term (therefore eligible) babies had EOGBS, a crude rate of 0.12/1000 term live births. All of these babies had mothers who were screened for GBS. These babies either had an eligible mother with a negative antenatal GBS screen (n = 7) or a mother with a positive GBS result that was unknown in labour and thus was unable to trigger IAP (n = 3). Six of the seven negative cultures were taken within five weeks of birth.

A further preterm baby, whose mother was eligible for GBS screening (≥ 35 weeks gestation) and had a positive result known in labour and was subsequently diagnosed with EOGBS.

Six of these 11 babies had additional risk factors documented (prematurity and/or maternal GBS colonisation, ROM ≥ 18 hours, fever), which would qualify for IAP using a risk-factor only approach.

Two mothers in the eligible group received some IAP, however neither of these women received a dose ≥4 hours before birth.

### Preterm babies with early-onset group B streptococcal infection

Eight babies with EOGBS were preterm (0.87/1000 preterm live births). Gestations ranged from 24 to 36 weeks. Two mothers in the preterm group received IAP (including the mother who was 36 weeks gestation and therefore eligible preterm), but neither dose was provided ≥ four hours before birth.

### Morbidity and mortality

Four of 10 term babies with EOGBS had serious short-term morbidity. All required neonatal intensive care and significant continuous positive airways pressure therapy, one baby had seizure activity. All four were discharged home in a well state and were recorded as living as of December 2017, as were the other six. One preterm baby born <32 weeks gestation died (0.11/1000 live born preterm births) a combined term and preterm crude fatality rate of 0.01/1000 live births.

## Discussion

This cohort study describes the management of GBS risk in our LHD in an era of universal screening and describes analysis of the rates and trends of EOGBS in an 11-year period in a diverse range of birth settings.

We found no evidence to conclude a difference in rates of EOGBS between women reported as screened or not screened for GBS. Our findings, along with others [32–34], highlight the logistical difficulty of mounting a sustained, consistent screening program across a large LHD. Furthermore, 12% of women who were not screened received IAP Table 2. The reasons for this were not analysed in this study.

Incidence of EOGBS in this population was low, at 0.19/1000 live births. The low frequency of EOGBS events limited our ability to explore time trends in incidence rates however our model did not provide evidence of a change in incidence over time. Our data align with the contemporary incidence rates recorded by a large, multi-centre study from the US, (0.2/1000 live births) [35].

It is possible and widely reported, that the rates of EOGBS may have remained low since the early 2000s because of screening and IAP provision [1, 34–36]. We found 21.5% of the 69% of pregnancies who were screened and had a result documented in the database, were positive for GBS. A modest 79% of women, who were positive for GBS at the end of their pregnancy, received some IAP Table 2. This finding is similar to the results found in a recent Australian integrative review [37]. The review found that although screening and IAP appeared to be very effective in reducing rates of EOGBS, the rate of IAP provision in the clinical setting was not optimal suggesting there may be other reasons for very low EOGBS rates. The database in our study did not specify the dose or frequency of IAP provision, so we could not establish if the IAP provided was assessed as adequate at a population level. At an individual level, to explore the experience of babies who had EOGBS, we examined individual medical records to obtain data not entered onto the ObstetriX database. None of the babies with EOGBS had mothers who were provided with adequate IAP.

In this era of universal screening and IAP provision there is no way of knowing what the rates of EOGBS in high income countries would be in babies whose mothers had GBS risk factors but no exposure to IAP. When data are compared from jurisdictions that use universal screening and IAP, versus a method based on risk factors, reported EOGBS rates are mixed, with either no change [25] increases in some jurisdictions [26,38] and decreases in others [21]. It should be noted, however, that some clinicians use a combination of the two standard methods of selecting women most at risk of having a baby affected by this infection [39] so comparison between countries, and even areas within countries, is problematic.

Seven out of the ten term babies with EOGBS had mothers who were screened negative for GBS. These data concur with others reporting on EOGBS in the era of widespread IAP provision, finding rates of infection occurring among babies born to women with pregnancies negative for GBS were higher than previously reported [35, 40]. There are several reasons why this may be the case. These false negative results may be due to the modest predictive values of current screening protocols; which are influenced by intermittent maternal GBS colonisation [26]. Further, in some jurisdictions, swabs may be incorrectly taken and /or transported, or incorrectly processed [14].

Five of the seven term women whose pregnancies screened negative for GBS had another risk factor for infection (ROM ≥18 hours) warranting consideration for IAP using a risk-based approach. Even though the most common risk factor for EOGBS in our study and others [41, 42] was ROM ≥18 hours, numbers were too small (5/7 term women) to draw any conclusion. IAP was not administered to the five women with ROM ≥18 hours and a negative GBS result, in accord with local guideline recommendations at the time. Early-onset infection due to GBS occurring in babies born to women whose pregnancies have screened negative for GBS, further reflects the limitations of current methods of assessing GBS risk in our area.

As well as the protocol of screening and IAP provision that was offered to most, but not all, women with pregnancies that had a GBS positive result, it is likely that the low rates of EOGBS in our cohort maybe related to other factors. It is true that the crude rates of maternal GBS colonisation in the cohort neither changed significantly from year to year nor materially between 2006 and 2016. However, shifts in GBS serotypes and/or virulence of the bacteria may have occurred. Furthermore, population differences in exposure to GBS, maternal immunity, and foetal/neonatal susceptibility may also play a role in the reduction of infection rates [43]. Our low incidence of EOGBS in term babies cannot be exclusively ascribed to the protocol of offering women universal GBS screening and IAP.

Term babies in our study, diagnosed with EOGBS, were promptly treated and all survived. Case fatality in preterm babies with EOGBS was 0.01/1000 preterm live births.

Reduction in mortality since the 1970s, which was then as high as half of both term and preterm babies with EOGBS, is thought to be largely due to advances in maternity and neonatal care [14].

### Limitations

Like many before, this study underestimates the true burden of EOGBS because it focuses on live born babies with culture-proven events, missing stillbirths and cases of clinical infection. Due to the rarity of EOGBS small case numbers prevented a more in-depth analysis; particularly of screened versus non-screened pregnancies.

We were unable to accurately record incidences of intrapartum fever and some other risk factors; a previous baby with EOGBS or bacteriuria in the index pregnancy and we were unable to access information for babies who were term and otherwise well but may have received antibiotics because of deemed inadequate chemoprophylaxis.

Our study uses a retrospective ascertainment of screening results, which may suffer from reporting bias. The high rate of undocumented GBS results in the database is a limitation and may be due, in part, to substandard data entry.

### Strengths

This retrospective study covering 11 years includes regional, rural and some remote birthing populations using a range of birthing options; from a metropolitan unit, to smaller regional units, birth centres and planned homebirth. Our study is generalisable to other jurisdictions with similar demographics and can be replicated in areas where researchers are able to collect pathology data and link these with a maternity and neonatal database such as ObstetriX or e. Maternity.

### Future direction

Three decades ago, IAP was introduced as a safe but interim solution to manage GBS risk [35]. Mortality rates since the 1970s have dropped markedly and EOGBS is now a rare and treatable infection, even in babies who are not exposed to IAP as this study and others report [35]. Since the introduction of universal screening and IAP, the number of women and babies exposed to prophylactic antibiotics in labour for GBS risk has more than doubled (12% to 30%) in some jurisdictions. Most term babies exposed to IAP have negligible risk of succumbing to the infection and are therefore, arguably, exposed to IAP unnecessarily. Intrapartum antibiotic provision is not without its own set of risks. There is emerging speculative data associating intrapartum antibiotics with adverse health issues later in life [5–9]. Interventions of any kind are likely to have wider effects than acknowledged by evaluators. For ethical and methodological reasons, it is imperative that any harmful effects of interventions as well as their short-term benefits, are considered, analysed and, if relevant, alleviated. Furthermore, if universal screening continues to be recommended and used widely, high quality research to assess the relative benefits and risks of a universal screening protocol versus a risk-based approach is warranted. This will provide clinicians, women, and their families’ access to high-quality evidence to enable them to discuss and make decisions about the risks they are prepared to embrace.

The potential for a maternal GBS vaccination to reduce the risk of EOGBS in term babies is supported by studies, which demonstrate that higher maternal serotype-specific antibody concentrations are associated with a lower risk of EOGBS. However, performing field trials on protein-conjugated GBS vaccines during pregnancy is not without its challenges, with large efficacy trials versus limited immunogenic studies being considered once a correlate of protection is universally identified and accepted [44].

Apart from vaccination, there may be other methods of reducing missed opportunities to provide IAP to those who would most benefit while reducing the number of mothers and babies unnecessarily exposed to IAP. Accurate and rapid methods of intrapartum GBS testing, aimed at a specific cohort of women who experience ROM without timely onset of labour, may assist in the identification of GBS status and assist women and clinicians in subsequent GBS risk management. To ensure optimum equity in maternity care, such a test should ideally be available to women accessing a variety of birth settings. In our LHD this proposal would require a point-of-care molecular test. Logistical and expense considerations may be challenging due to the wide variety of birth settings in our region. Point-of-care testing, however, has been offered for some time at a large metropolitan hospital in an adjoining LHD and may reduce the number of women and babies at term gestation unnecessarily exposed to intrapartum antibiotics.

Following presentation of this study, decision makers in our LHD have resolved not to increase the dose of prophylactic antibiotics for maternal GBS colonisation due to our very low and stable term EOGBS rates. This HNELHD will therefore not be in line with the current Australian therapeutic guidelines and the CDC recommendations for prophylaxis, which we believe, warrant review.

Based on the results of this study we, along with others [45], strongly recommend that primary attention to risk factors for EOGBS infection and timely prophylaxis or antibiotic treatment as indicated would be a more effective strategy for reduction of EOGBS in both preterm and term groups rather than the universal screening approach which failed to identify all infants at risk.
